# Research on soybean leaf disease recognition in natural environment based on improved Yolov8

**DOI:** 10.3389/fpls.2025.1523633

**Published:** 2025-04-07

**Authors:** Chen Chen, Xiaolei Lu, Lei He, Ruoxue Xu, Yi Yang, Jing Qiu

**Affiliations:** ^1^ College of Big Data (College of Information Engineering), Yunnan Agricultural University, Kunming, China; ^2^ The Key Laboratory for Crop Production and Smart Agricultural of Yunnan Province, Yunnan Agricultural University, Kunming, China; ^3^ School of Information and Intelligent Engineering, Yunnan College of Business Management, Kunming, China

**Keywords:** soybean leaf disease, attention mechanism, multi-scale features, object detection, YOLOv8

## Abstract

The rapid and accurate identification of soybean diseases is critical for optimizing both yield and quality. Traditional image recognition techniques face notable limitations in terms of generalization and accuracy, particularly when tasked with identifying small-scale targets or distinguishing diseases with similar characteristics in large, heterogeneous, and complex environments. To address these challenges, this study proposes the YOLOv8-DML model for soybean leaf disease recognition. Building upon YOLOv8n, this model integrates a DWR module that replaces the high-level C2f module with C2f-DWR, enhancing feature extraction across varied receptive fields. Additionally, modifications to the neck structure incorporate a Multi-scale Enhanced Feature Pyramid (MEFP), which improves detection performance across targets of varying sizes by enabling effective multi-scale information fusion. A lightweight detection head (LSCD) is further introduced to facilitate multiscale feature interactions while reducing the overall model parameter count. Lastly, the WIoUv3 loss function is employed to place greater emphasis on small targets and moderate-quality samples, thereby enhancing detection precision. Experimental results demonstrate that YOLOv8-DML achieves a mAP50 of 96.9%, marking a 1.8% improvement over the original YOLOv8 algorithm, while also achieving an 18.6% reduction in parameters. Comparative analysis with other mainstream object detection models indicates that YOLOv8-DML delivers superior overall performance, highlighting its significant potential for effective soybean leaf disease identification.

## 1 Introduction

As one of the world’s most crucial food crops, soybeans are rich in plant proteins and play a key role in both food and processing industries (Jianing et al., 2022). In 2023, global soybean production reached 318 million tons, with China contributing only 20.84 million tons, or 6% of the global output. China’s production falls far short of meeting the domestic demand, with an average annual soybean consumption of 100 million tons. Surveys indicate that soybean yield losses due to diseases account for 10%-30% of the total annual production annually (Chang et al., 2017; Guo et al., 2021). Soybean leaf diseases are major limiting factors for both yield and quality (Peng et al., 2021). Effective disease control requires targeted intervention in the early stages (Huang et al., 2014). Traditional disease identification methods primarily rely on the subjective judgment of experienced farmers or plant protection experts. This approach not only has low efficiency but also struggles to ensure accuracy (Dhakal and Shakya, 2018). When identification relies solely on manual experience, diseases often miss optimal prevention and control periods, as early-stage symptoms are typically subtle. Thus, the early and accurate identification of soybean leaf diseases is critical for effective management and high-quality production. Advances in machine learning and neural networks have enabled the application of numerous algorithms to plant leaf disease identification. Currently, plant disease identification primarily utilizes two approaches: traditional machine learning and deep learning-based methods.

Currently, few studies have explored soybean leaf disease recognition using deep learning. Most studies have focused on soybean leaf disease images captured in controlled environments. These models often lack generalization in complex natural settings and perform suboptimally when recognizing leaf diseases with similar features. Additionally, the variety of soybean leaf diseases, combined with environmental and growth-stage variations, results in substantial differences in color, morphology, distribution, and lesion size. Lesions of varying sizes appear on the same leaf, with some being small and densely clustered, which increases the complexity of disease recognition and hinders the learning of small lesion features. This necessitates deep learning models that possess heightened sensitivity and precision for feature extraction. To address these challenges, this study proposes YOLOv8-DML, a soybean leaf disease recognition model based on an improved version of YOLOv8, designed specifically for natural environments. The main contributions of this study are as follows.

1. Multi-source heterogeneous data fusion strategy: To enhance the robustness and generalization of the model, this study integrates public datasets (iBean and Digipathos) with a self-collected soybean leaf disease dataset from Baoshan, Yunnan, containing 1,581 images across four diseases (soybean angular leaf spot, soybean anthracnose, soybean rust, and soybean yellow mosaic) and healthy leaves. Additionally, diverse data augmentation techniques have been employed to expand the image and label data, thereby enhancing the adaptability of the model to various environments and conditions.
2. Model enhancement for detection performance: This study proposed the YOLOv8-DML model for soybean leaf disease recognition, incorporating C2f-DWR and MEFP modules to improve the detection capability across diseases of varying scales. Additionally, a lightweight detection head (LSCD) was designed to optimize the feature extraction and detection efficiency and reduce the computational cost while maintaining high-precision detection. Finally, WIoUv3 was employed as a loss function to further enhance the model accuracy.
3. Model’s actual recognition performance: The experimental results indicate that YOLOv8-DML significantly outperforms the other models in terms of soybean leaf disease recognition accuracy. It notably enhances the detection of densely distributed, multi-scale diseases and enables effective recognition of soybean leaf diseases in natural environments.

The remainder of this paper is organized as follows: Section II summarizes the domain survey.Section III details the dataset construction and preprocessing methods, including multi-source heterogeneous data fusion and data enhancement techniques. Section IV covers the design and enhancement of the YOLOv8-DML model. Section V presents the experimental results, provides a comprehensive analysis, and validates the model performance through multiple comparative experiments. Section VI concludes with a summary and discussion of the research findings.

## 2 Related works

Feature extraction and classification using traditional machine learning algorithms represent conventional approaches for plant leaf disease classification. These methods offer the advantages of rapid recognition and minimal hardware requirements. Pujari et al. (2016) extracted color and texture features from plant leaf images, employing feature selection methods to reduce dimensionality. Subsequently, two classifiers, Support Vector Machine (SVM) and Artificial Neural Network (ANN), were used to train and test the features, and their performances were compared. The experimental results indicated that the SVM classifier achieved higher accuracy in plant disease recognition, with an average accuracy of 92%. Sharif et al. (2018)] proposed a machine learning-based approach for citrus disease detection and classification. Initially, a weighted segmentation method was applied to extract disease spots, followed by the fusion of color, texture, and geometric features into a codebook, which was then input into an SVM for final classification. Mondal et al. (2017) extracted 43 features from leaf images and applied the Pearson correlation coefficient method for key feature selection. An entropy-based discretization method and a Naive Bayes classifier were then employed to classify Yellow Vein Mosaic Virus disease. Xie et al. (2017) utilized the K-Nearest Neighbors (KNN) algorithm to classify healthy and diseased tomato leaves affected by gray mold, enabling early detection. Gold et al. (2020) employed reflectance spectroscopy and machine learning to investigate physiological differences among potato varieties in response to late blight infection. Using statistical methods, including Random Forest (RF), the study revealed that potato variety significantly affects spectral reflectance, and that different varieties exhibit varied responses to pathogens at different infection stages. Although traditional machine learning algorithms have yielded notable results in crop disease identification, they often exhibit limitations when applied to images of complex diseases. First, the feature extraction step is critical; however, it typically relies on expert knowledge, making it highly subjective and constrained. Moreover, feature selection and model training require optimization for each disease, limiting their adaptability to multiple diseases across diverse environments. Additionally, traditional machine learning methods have limited generalization capabilities, particularly when dealing with large and diverse datasets.

In recent years, deep learning has demonstrated significant potential in image classification and recognition. Unlike traditional methods, deep learning automatically extracts features and achieves more accurate classification via multi-layer neural networks. This approach reduces reliance on manual feature extraction and enhances generalization capability through large-scale data training. This technology has been widely applied to crop disease identification (Afifi et al., 2020; Anitha and Srinivasan, 2022; Elaraby et al., 2022). Mao et al. (2022) developed an enhanced regional convolutional neural network that significantly improved recognition accuracy for wheat stripe rust and yellow dwarf disease, enabling early detection. However, yellowing symptoms from other causes were not categorized, potentially limiting the generalizability of the model. Zhang et al. (2021) applied the Faster R-CNN model with multi-feature fusion to distinguish characteristics of soybean leaves, including healthy, diseased, and variably affected leaves. The test achieved an average accuracy of 83.34%. Haque et al., (2022a, b); Haque et al. (2023) conducted experiments on common corn diseases using GoogLeNet and Inception V3, designing a novel CNN network to classify and assess disease severity. The proposed method yielded favorable results in identifying common corn diseases and made notable progress in model lightweight. Srilakshmi and Geetha (2023) introduced a DIM-U-Net method based on SR-AE and LSTM, applying it to leaf classification. Through metrics such as accuracy, sensitivity, specificity, precision, F1 score, and AUC, this method provides valuable guidance for the accurate detection and classification of soybean leaf diseases. Yu et al., (2022; 2023) applied the OTSU algorithm to remove background influence from soybean leaf disease images, isolating single-leaf disease representations. A residual attention layer (RAL) was constructed within ResNet18 using shortcut connections, replacing its residual structure and transferring weights from the pre-trained convolutional layer to ResNet18. New residual attention networks, RANet and TRNet18, have been developed to enable the accurate, rapid, and efficient recognition of soybean leaf diseases. Wu et al. (2023) proposed a soybean leaf disease classification method integrating ConvNeXt with an attention module. This method captures attention feature maps at various network depths via the CBAM module, employing the LeakyReLU activation function to prevent neuron failure during training, thus enhancing classification accuracy. The experimental results indicate that the improved ConvNeXt model achieves high accuracy in soybean leaf disease recognition under complex backgrounds. Kaler et al. (2023) employed deep learning architectures, including long short-term memory (LSTM), neural networks (NN), convolutional LSTM (Conv LSTM), and three-dimensional CNN (3D CNN), to detect soybean leaf diseases in complex environments.

Hyperspectral and multispectral imaging technologies are recognized as effective tools for disease detection due to their ability to capture rich spectral information that distinguishes healthy from diseased plant tissues. Hyperspectral imaging provides a detailed spectral profile for each pixel, enabling precise identification of disease-related biochemical changes, while multispectral imaging offers a simplified yet practical approach for real-time applications in agriculture. Studies have demonstrated the effectiveness of hyperspectral imaging in identifying soybean diseases. For example, Zhang et al. (2020) developed a hyperspectral imaging system for the early detection of soybean rust, achieving high classification accuracy by analyzing spectral reflectance features. Similarly, Li et al. (2019) proposed a multispectral imaging method to distinguish diseased soybean leaves using selected key wavelengths, which improved detection efficiency while reducing data redundancy. Furthermore, Mahlein et al. (2018) reviewed the application of optical sensors, including hyperspectral and multispectral technologies, in plant disease detection, emphasizing their potential for accurate and non-invasive monitoring of plant health. These studies highlight the importance of spectral wavelength analysis for disease detection, underscoring the need to incorporate spectral information into disease identification models Zhang et al. (2021). However, compared with visible light images, high-frequency spectral images are more difficult to collect, so the application is limited.

## 3 Materials and methods

### 3.1 Dataset construction

To enhance practical applicability, this study adopted a multi-source heterogeneous data fusion strategy that integrated soybean leaf disease images from various regions, varieties, and growth stages to construct a comprehensive soybean disease dataset.

The disease dataset utilized in this study was composed of three primary sources, providing a diverse and comprehensive collection of soybean leaf images:

1. Custom Soybean Leaf Disease Dataset: This dataset was collected in Wuding County, Chuxiong Prefecture, Yunnan Province, using an iPhone 13. The dataset features high-resolution images captured under natural lighting conditions, representing various stages of soybean disease progression. Efforts were made to include leaves with diverse morphological characteristics, ensuring that the dataset reflects a wide range of real-world conditions encountered in soybean fields.
2. iBean Dataset: Developed by the Makerere AI Laboratory in collaboration with the National Crop Resources Institute (NCRI) of Uganda, this dataset consists of a large collection of soybean leaf images sourced from multiple regions across Uganda. The images were captured in natural field environments, incorporating variations in lighting, disease progression stages, and plant morphology. This diversity makes the iBean dataset a valuable resource for evaluating the robustness of detection models across geographically and environmentally distinct regions (Ernest, 2020).
3. Digipathos Dataset: Provided by the Brazilian Agricultural Research Agency (Embrapa), this dataset includes soybean leaf images captured under field conditions in Brazil. The dataset emphasizes disease progression in natural environments, offering insights into how diseases manifest under different environmental and climatic conditions. As with the other datasets, images feature variations in leaf morphology, lighting, and disease development stages (Barbedo et al., 2018).
4. These datasets collectively encompass images of four common soybean diseases: angular leaf spot, anthracnose, rust, and yellow mosaic disease, in addition to healthy soybean leaves. In total, the dataset comprises 1,581 annotated samples, providing a robust foundation for training and evaluating the proposed model. Each image captures the natural progression of diseases, accounting for factors such as varying illumination, diverse leaf shapes, and different stages of disease development, ensuring the dataset’s applicability to real-world scenarios.

**Figure 1:**
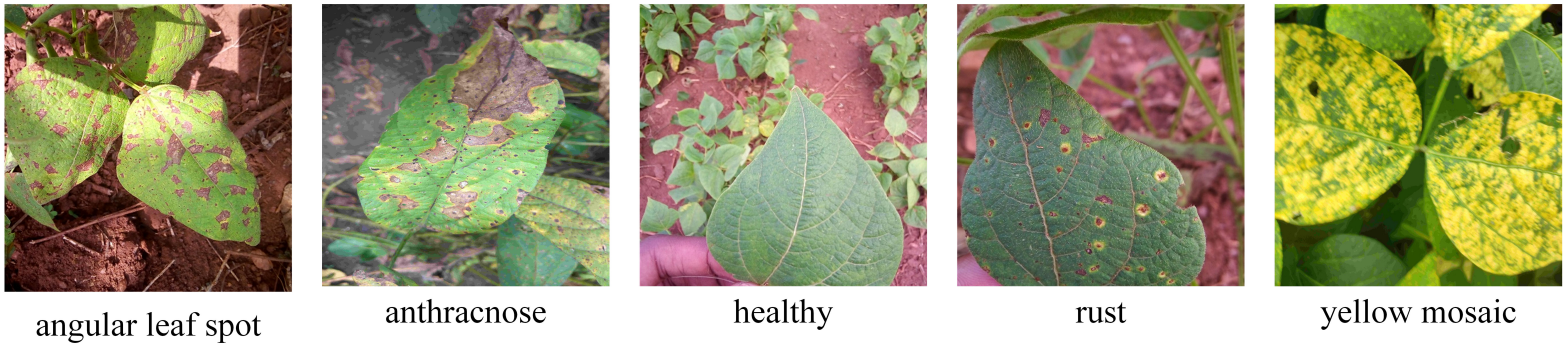
Examples of three soybean leaf disease image datasets used in this paper. Some images are sourced from public datasets (Ernest, 2020; Barbedo et al., 2018).

**Figure 1** illustrates representative samples from the compiled soybean disease dataset, highlighting the variations in disease symptoms and leaf morphology across the different datasets. By integrating datasets from geographically distinct regions with diverse environmental conditions, this study ensures that the proposed model is not only accurate but also generalizable across a wide range of natural scenarios.

### 3.2 Image data preprocessing

**Figure 2:**
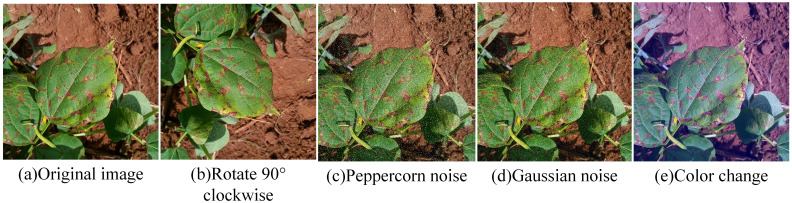
Example of image enhancement. **(a)** Original image; **(b)** Rotate 90° clockwise: **(c)** Peppercorn noise; **(d)** Gaussian noise; **(e)** Color change.

To mitigate model overfitting or underfitting due to insufficient or imbalanced sample sizes, this study applied various image enhancement techniques, including rotation, salt-and-pepper noise, Gaussian noise, and color adjustment, to augment the original dataset, thereby improving model generalization and preventing overfitting. After enhancement, the disease image dataset comprised 18,875 samples. To facilitate training, the images were normalized and resized to 640×640 pixels. Labels were assigned to the soybean image dataset using the values 0, 1, 2, 3, and 4 to denote the respective categories. The dataset was saved in JPEG format and imported into the system to construct a soybean leaf disease database. The dataset was divided into training, validation, and test sets in a 7:2:1 ratio. An example of image enhancement is shown in **Figure 2**. The distribution of the post-enhancement soybean leaf disease images is shown in **Table 1**.

**Table 1:** Distribution of soybean leaf disease dataset.

Category	Name	Original images	After data enhancement	Training set	Validation set	Test Set
0	angular_leaf_spot	345	4140	2892	824	424
1	anthracnose	218	3186	2211	659	316
2	healthy	340	5100	3616	998	486
3	rust	348	3480	2418	699	363
4	yellow_mosaic	330	2969	2075	595	299
Total number of images		1581	18875	13212	3775	1888

## 4 Construction of soybean leaf disease identification model

### 4.1 YOLOv8 model

The YOLOv8 model, developed and maintained by startup Ultralytics, includes five versions: YOLOv8n (Nano), YOLOv8s (Small), YOLOv8m (Medium), YOLOv8l (Large), and YOLOv8x (Extra Large). The model architecture is primarily divided into three modules: backbone, neck, and head networks.

The backbone network of YOLOv8 functions as a feature extractor and is typically built upon convolutional neural network (CNN) architectures like Darknet or CSPDarknet. Its primary role is to extract meaningful features from input images for further processing. By applying a series of convolutional and pooling operations, the backbone progressively reduces the spatial dimensions of feature maps while increasing their depth, enabling it to effectively capture and represent different levels of complexity and scale in the input image (Chai et al., 2021).

The neck network employs a hybrid structure of a Feature Pyramid Network (FPN) and a Path Aggregation Network (PAN). Its primary purpose is to integrate feature information from different levels to enhance object detection performance. The neck network includes multiple operations, such as convolution, upsampling, and downsampling, designed to merge feature maps at different resolutions and enhance the network’s ability to capture contextual information and target details (Terven et al., 2023).

The architecture of YOLOv8’s head section utilizes a decoupled structure, effectively separating the classification and detection components. Two parallel branches are employed to extract categorical and positional features, which are then processed using a 1×1 convolutional layer to perform classification and localization tasks. Additionally, YOLOv8 integrates an adaptive anchor box mechanism that generates anchor boxes tailored to the shapes and sizes of the targets, accounting for the variability in the dataset. This approach improves the bounding box regression process, leading to better detection accuracy. Regarding the loss function, YOLOv8 adopts a comprehensive optimization strategy that combines classification, regression, and target confidence loss, while incorporating the CIoU loss to refine bounding box fitting and ensure reliable detection results.

Given the extensive receptive field of the YOLOv8 model, its accuracy proves inadequate for small target positioning, resulting in suboptimal detection performance. Furthermore, the center point-based Anchor-Free detection algorithm employed by the model exhibits limitations in both center point offset and target size prediction when addressing lesions of varying shapes and sizes. The algorithm is susceptible to interference from neighboring targets, particularly in regions characterized by substantial scale variations and high target density, which leads to positioning inaccuracies. Soybean leaf diseases exhibit considerable heterogeneity in their morphological characteristics, including shape and size, with numerous densely clustered small target lesions present. Employing the YOLOv8 model for soybean disease identification may result in unsatisfactory disease recognition outcomes primarily because of the inherent limitations of the model in processing small and dense targets.

### 4.2 YOLOv8-DML model

**Figure 3:**
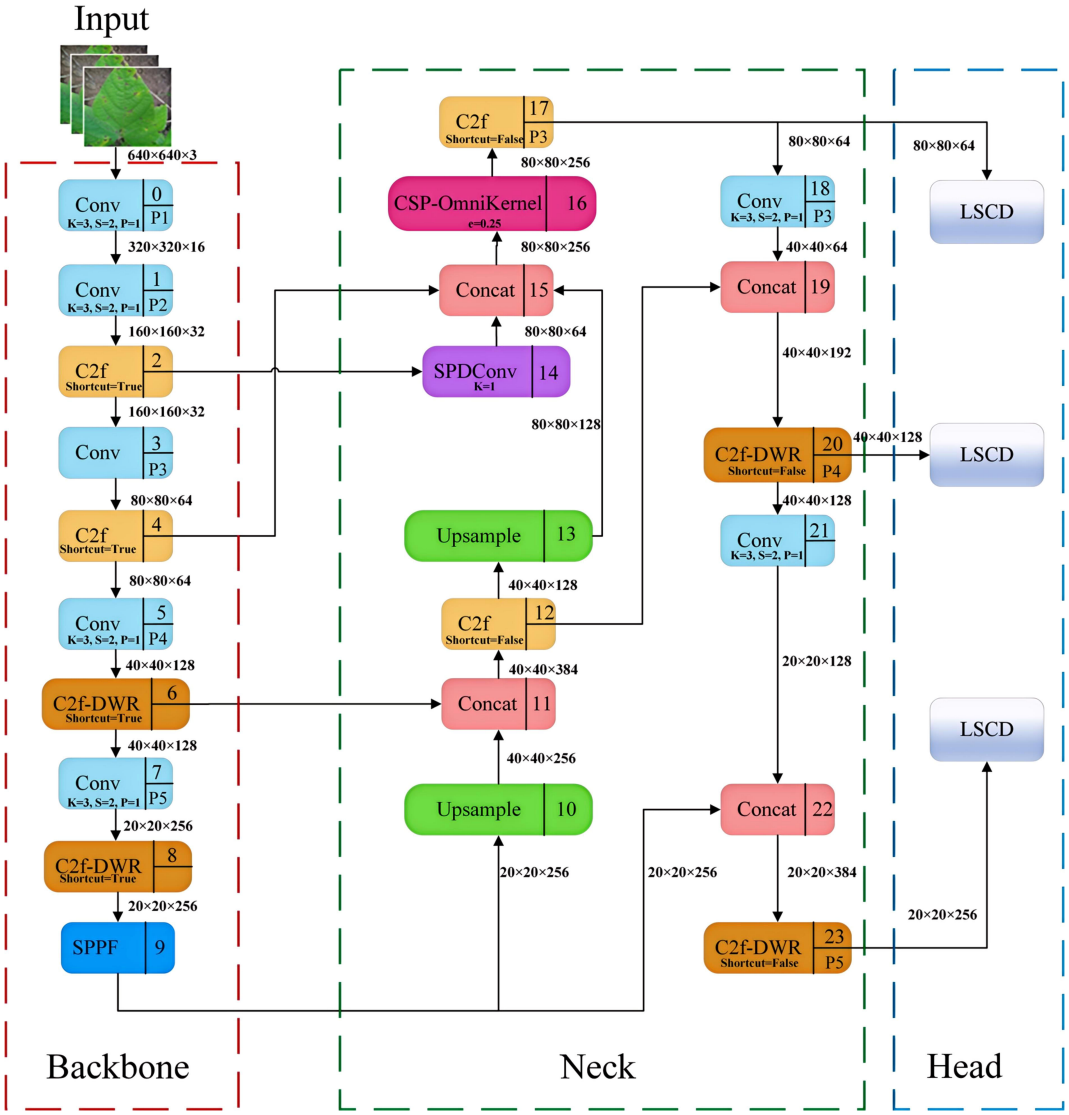
YOLOv8-DML model structure diagram.

To achieve a more precise identification of soybean leaf diseases in natural environments, this study developed a YOLOv8-DML model derived from YOLOv8n, aimed at enhancing the model’s feature extraction and fusion capabilities, ultimately improving the recognition accuracy of soybean leaf diseases. The structure of the YOLOv8-DML model is illustrated in **Figure 3**.

Specifically, the YOLOv8-DML model enhanced the following aspects compared to the original model:

1. Backbone: The DWR module was integrated to augment the C2f architecture, resulting in the construction of the C2f-DWR module, which replaces the C2f component within the P4 and P5 feature layers of the backbone network. This modification effectively expands the receptive field of the model through a multi-branch dilated convolution structure, thereby enhancing the deep feature extraction capabilities. Furthermore, it significantly optimizes the model for multi-scale feature learning and improves its generalization performance.
2. Neck: The original PAFPN was further enhanced, culminating in the design of the Multi-scale Enhanced Feature Pyramid (MEFP). This modification incorporates feature maps from the P2 layer, and integrates the SPDConv, CSP-OmniKernel module, and C2f-DWR module, thereby substantially improving the model’s global and local feature representation across varying scales. This enhancement not only boosts detection performance but also improves the model’s ability to recognize multi-scale targets. Additionally, the dynamic adaptation capacity of the OmniKernel module, combined with the efficient gradient flow characteristic of the CSP structure, enables more effective multi-scale feature extraction and integration, all while maintaining low computational complexity.
3. Head: A lightweight shared convolution detection head (LSCD) was developed, incorporating two weight-shared DEConv modules. This design significantly reduces the computational complexity of the YOLOv8 decoupled head while improving the model’s ability to fuse multi-scale features and enhance its representational power. Moreover, the integration of group normalization and a dynamic scale adjustment module improves feature stability and detection accuracy across targets of varying scales, thereby achieving high-accuracy performance with a minimal number of parameters.
4. Loss Function: The WIoUv3 loss function was selected to replace CIoU in YOLOv8, incorporating a dynamic non-monotone focusing coefficient. This adjustment enhances the model’s sensitivity to irregular small lesions, optimizing its focus on medium-quality anchor frames. As a result, the loss function significantly enhances the robustness and overall performance of the disease detection process.

### 4.3 C2f-DWR dilated residual module

**Figure 4:**
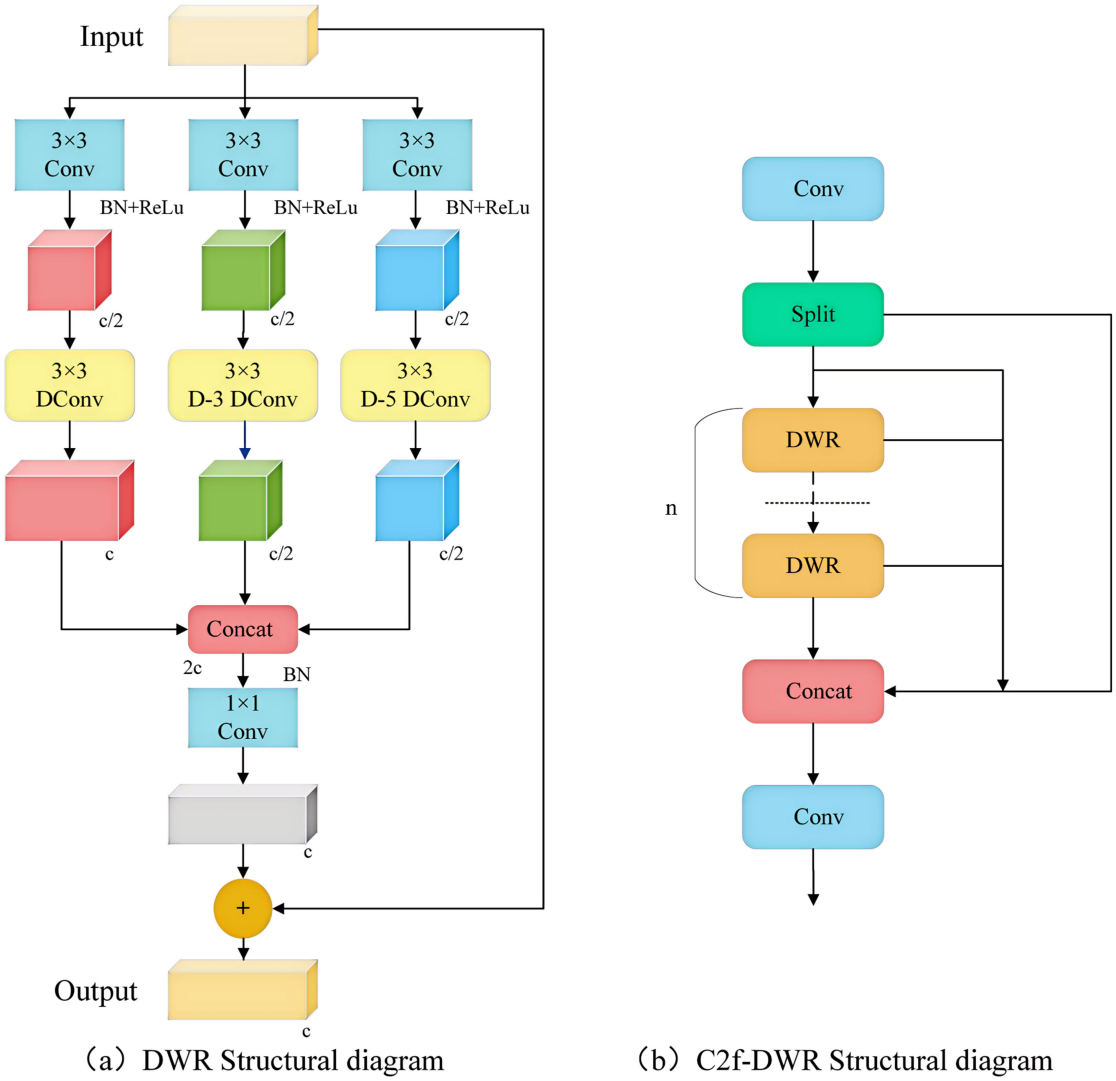
DWR and C2f-DWR structure diagram. **(a)** DWR Structural diagram; **(b)** C2f-DWR Structural diagram.

The receptive field size of the convolutional layer within the C2f module of the YOLOv8 model is static, which complicates the effective acquisition of global information. Although it is possible to enlarge the receptive field by stacking multiple convolutional layers, this approach significantly increases both computational cost and model complexity. Consequently, the DWR module was introduced to develop the expanded residual module C2f-DWR to enhance the deep feature extraction capabilities. The DWR module is a multi-branch architecture, as illustrated in **Figure 4a**. The DWR module initially employs a 3×3 convolution to extract feature information and subsequently utilizes three branch structures to expand the receptive field. Each branch conducted 3×3 depth-wise convolutions with expansion rates of 1, 3, and 5 to capture semantic information. Finally, the feature map derives semantic residuals through a Batch Normalization (BN) layer and employs a 1×1 convolution to extract spatial features.

By integrating multiscale features with residual learning strategies, the Bottleneck in C2f was replaced by a DWR module, resulting in the construction of a new module, C2f-DWR. The C2f-DWR module employs deep convolution with varying dilation rates to expand the receptive field of the model, enabling it to adaptively capture target features at different scales in the disease images, thereby enhancing the model’s generalization capability. **Figure 4b** illustrates the structure of the C2f-DWR module. The C2f-DWR module further optimizes the model’s performance in multiscale feature learning while preserving the efficient characteristics inherent to C2f.Multi-scale Enhanced Feature Pyramid (MEFP).

Soybean horn leaf disease, rust, and similar conditions typically manifest as small spots. Compared to larger surrounding targets, such as soybean anthracnose, yellow leaf disease, and healthy leaves, these spots are often inconspicuous and easily overlooked. The original architecture of YOLOv8 comprises three detection heads (P3, P4, and P5). Given that downsampling results in the loss of small target information, the feature extraction effectiveness for small targets in the P3, P4, and P5 layers may prove inadequate. The P2 feature layer incorporates fine-grained features and edge information, thereby facilitating the extraction of small target features. A common practice involves adding a P2 detection layer to the model to enhance the detection capability for small targets; however, this approach increases both the computational load and the processing time.

**Figure 5:**
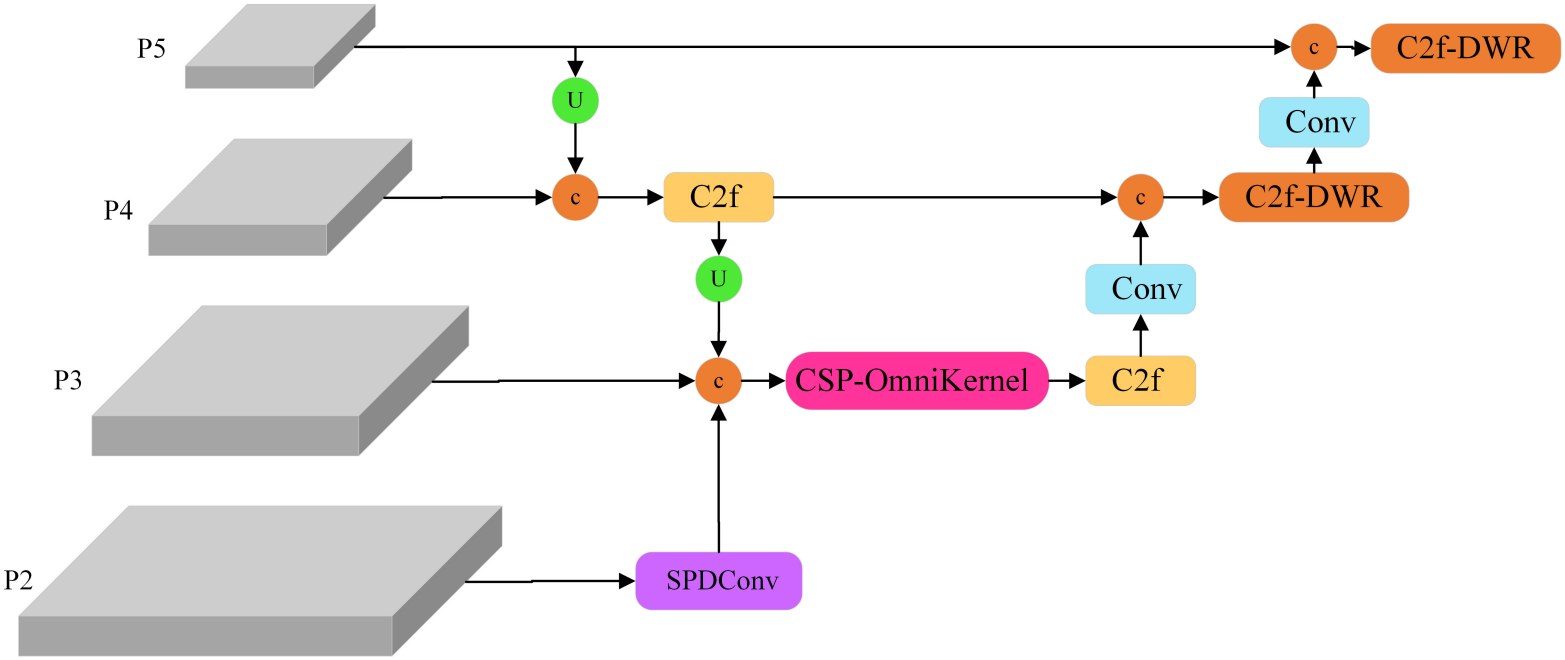
MEFP structure diagram.

This study develops a Multi-Scale Enhanced Pyramid (MEFP), the structure of which is illustrated in **Figure 5**. The original model’s network output feature maps measured 20×20, 40×40, and 80×80, whereas the MEFP significantly enhanced the model’s capacity to extract global features by incorporating a 160×160 feature map output from the P2 layer. Specifically, the P2 feature layer undergoes processing via SPDConv to acquire scale features that are abundant in small target information. The extracted image features are subsequently fused with the P3 layer and the upsampling layer, followed by multi-scale feature integration through the CSP-OmniKernel module. The P4 and P5 layers were processed using the C2f-DWR module, which extracts global features through multi-expansion rate convolution kernels. This approach facilitates deeper feature learning, significantly enlarges the receptive field of the model, and enhances the detection capability for targets of various scales. Through these enhancements, the MEFP effectively learns feature representations that transition from global to local contexts, bolstering its capacity to extract global features and fuse multi-scale features, thereby improving the recognition capability for targets of various scales.

**Figure 6:**
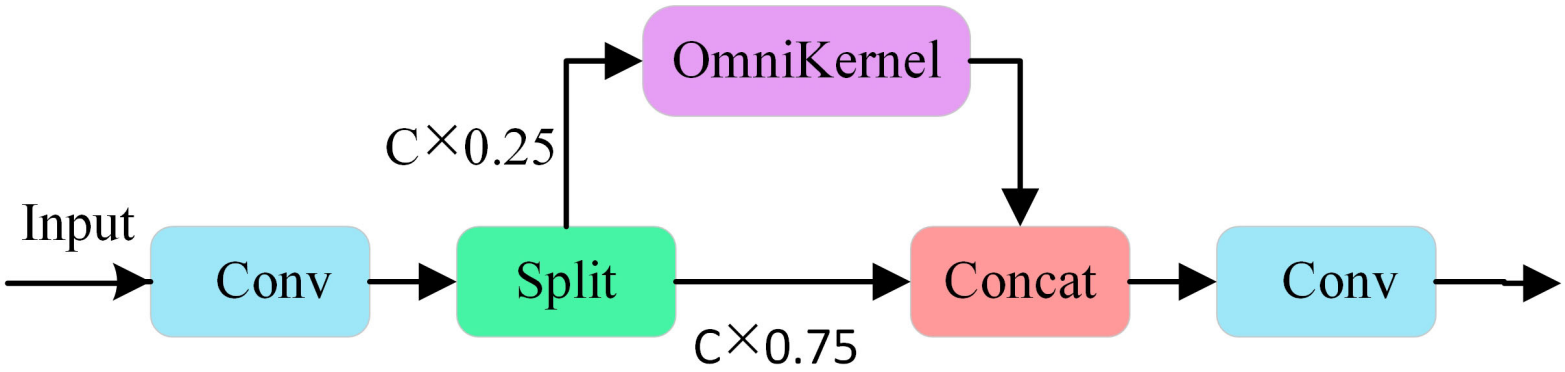
CSP-OmniKernel structure diagram.

The CSP-OmniKernel module integrates the structural concepts of the CSP with those of the OmniKernel module. Its structure is illustrated in **Figure 6**. The CSP structure effectively enhances the feature expression capabilities and improves the gradient fluidity by integrating certain features across stages. The OmniKernel module transcends the constraints of traditional fixed-size convolution kernels by enabling dynamic adaptation of the receptive field, thereby efficiently capturing multi-scale features with minimal computational overhead. The structure comprises three branches: a global branch, a large branch, and a local branch. The three branches collaboratively enhance multi-scale feature representation, ultimately achieving additional fusion, followed by further integration via the 1×1 convolution layer. In the processing flow of the input image features, the features undergo preliminary processing via 1×1 convolution, after which the channels are divided into two parts: 25% of the channels are processed by the OmniKernel module, while 75% remain unchanged. Subsequently, the two feature components are fused and passed through a convolution layer to produce the final enhanced feature map. This design fully utilizes the multiscale feature extraction capabilities of the OmniKernel module to capture target information across different scales, thereby enhancing the capacity of the model to detect targets of various sizes. Additionally, when combined with the characteristics inherent to the CSP concept, it effectively reduces the computational demands and model complexity, thereby further enhancing the recognition efficiency of the model.

### 4.4 LSCD detection head

**Figure 7:**
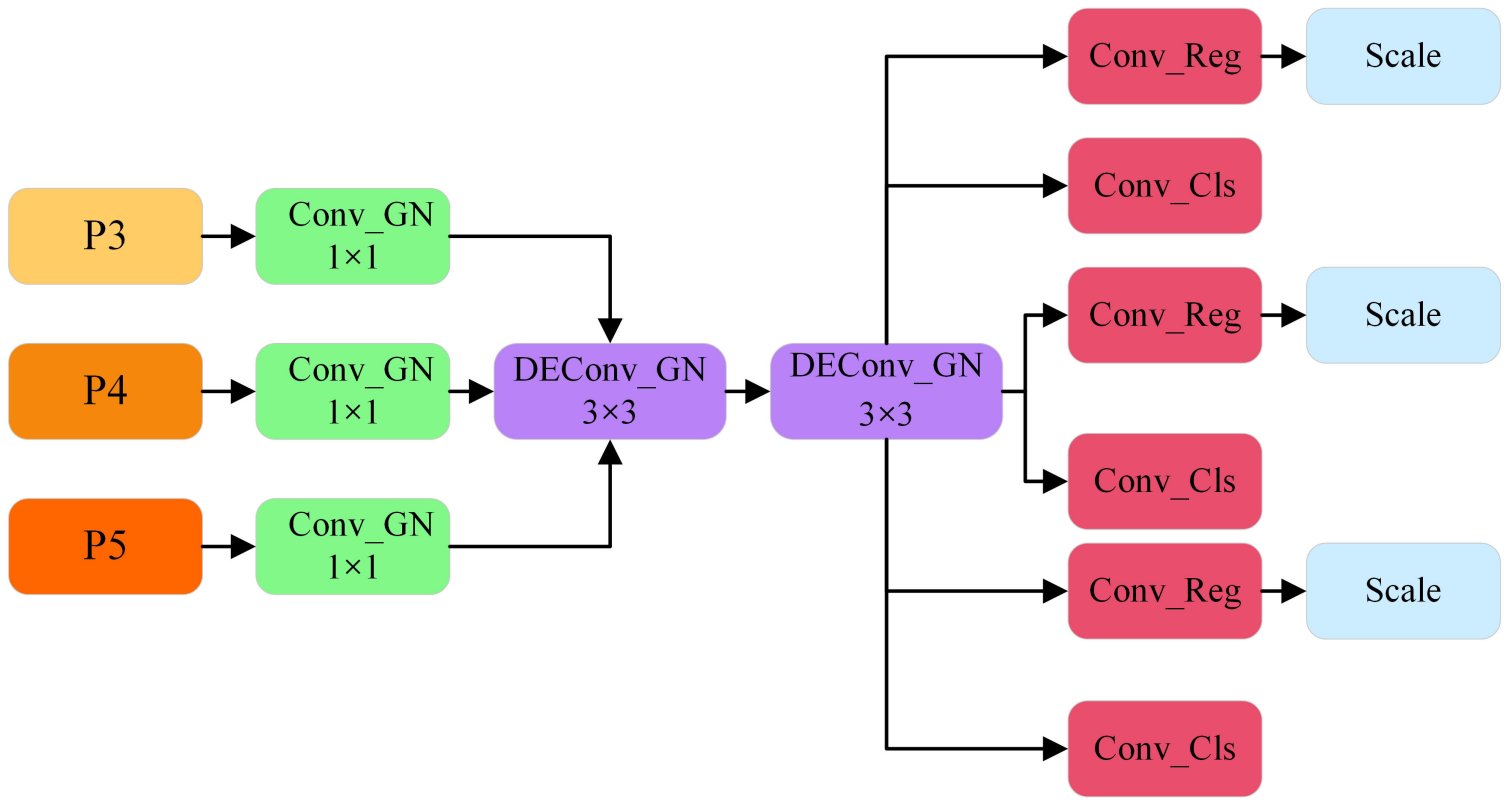
LSCD structure diagram.

YOLOv8 utilizes a decoupled head architecture that distinctly separates the classification and detection tasks. While this design enhances detection accuracy, it simultaneously imposes a substantial computational burden. To address this challenge, the present study introduces the Lightweight Shared Convolutional Detection Head (LSCD). By leveraging the weight-sharing DEConv module, the fusion of three feature maps at different scales is effectively achieved, thereby reducing the computational overhead. This method not only decreases the number of parameters but also alleviates the overall computational complexity. Moreover, the DEConv module incorporates prior knowledge into conventional convolutional layers, thereby significantly improving the model’s representational capacity and generalization performance. Simultaneously, by employing re-parameterization technology, DEConv is equivalently transformed into a conventional convolution layer without incurring additional computational costs, thereby ensuring that the model retains the same computational efficiency and compatibility as conventional convolution layers during the inference stage (Chen et al,. 2024). Additionally, Group Normalization (GN) is introduced to augment the positioning and classification accuracy of the detection head. This design enhances the extraction of detailed information from feature maps and facilitates information sharing across different detection layers, thereby reducing the number of parameters while preserving accuracy. Finally, a Scale layer was incorporated following each regression branch to dynamically adjust the target scale, addressing the challenge of inconsistent target sizes detected by each detection head. Its structure is illustrated in **Figure 7**.

The Lightweight Shared Convolutional Detection Head (LSCD) receives feature maps from the P3, P4, and P5 levels of the feature pyramid network as input, comprising three distinct layers. Each input feature map is initially processed through a 1×1 convolutional layer to standardize feature sizes across the layers. This convolutional operation is accompanied by Group Normalization, which not only standardizes the feature distribution but also mitigates the limitations of Batch Normalization in scenarios involving small batch sizes. As a result, stable feature representations are maintained across varying levels of the feature maps. Following this channel adjustment, two 3×3 DEConv layers are applied to extract multi-scale contextual information, facilitating the aggregation of richer spatial details. The shared weight design significantly reduces the number of parameters while ensuring contextual consistency across the feature maps. These feature maps are subsequently passed to the classification and regression branches for task decoupling. The classification branch is dedicated to target category identification, while the regression branch is responsible for spatial localization and scale adjustment. To further enhance the model’s performance, a dynamic scale adjustment module (Scale layer) is integrated into the regression branch. This module adaptively adjusts the scale of the target during the prediction phase, addressing scale discrepancies among targets processed by different detection heads. Such a design greatly improves the adaptability of the regression branch to targets of varying scales, thereby contributing to enhanced detection accuracy.

WIoU_V1_ modifies the penalty for bounding boxes by incorporating an attention mechanism, thereby augmenting the focus of the model on bounding boxes of ordinary quality (Tong et al., 2023). The calculation formula is presented in Equation 1. R_WIoU_ can markedly enhance the L_WIoU_ of ordinary quality anchor boxes, whereas L_WIoU_∈[0,1] significantly diminishes the R_WIoU_ of high-quality anchor boxes.


(1) $$L_{WIOU\;v1\;}=R_{WIOU\;}L_{IOU\;}$$

(2) $$R_{WIoU}=\text{exp}(\frac{{\left(x-x_{gt}\right)}^{2}+{\left(y-y_{gt}\right)}^{2}}{{\left(W_{g}^{2}+H_{g}^{2}\right)}^{*}}$$

Where L_WIoU_ represents the similarity or error between the detection box and the true box, and R_WIoU_ is the penalty term. The calculation formula of RWIoU is prsented in Equation 2. x and y represent the coordinates of the center point of the predicted box, $x_{gt}$ and $y_{gt}$ represent the coordinates of the center point of the true box, and. $W_{g}$ .and $H_{g}$ represent the width and height of the minimum bounding box, respectively.

WIoU_V2_ incorporates a monotonic clustering coefficient that effectively mitigates the adverse impact of low-quality samples on loss value (Tong et al., 2023). However, the incorporation of the focusing coefficient influences the reverse gradient behavior. Consequently, the exponential sliding average of L_IoU_ is utilized as the normalization factor to address the issue of diminished gradient gain resulting from the decrease in L_IoU_ The revised calculation formula for WIoU_V2_ is presented in Equation 3.


(3) $$L_{WIoUv2}={\left(\frac{L_{IoU}^{*}}{\bar{L_{IoU}}}\right)}^{\gamma }L_{WIoUv1}$$

Where. $\frac{L_{IoU}^{*}}{\bar{L_{IoU}}}$ . represents the gradient gain r, and $\bar{L_{IoU}}$ is the exponential running average with momentum m.

WIoUv3 constructs a dynamic, non-monotonic focusing coefficient through the introduction of the outlier *β*, which diminishes the gradient gain of high-quality anchor boxes while concurrently suppressing the detrimental gradients produced by low-quality samples (Tong et al., 2023). This mechanism allows the model to focus more on medium-quality anchor boxes, thereby preventing the over-optimization of both high-quality and low-quality samples, which effectively enhances the overall performance of the model. The calculation formula for this mechanism is presented in Equation 4:

$\beta =\frac{L_{IoU}^{*}}{\bar{L_{IoU}}}\in \left[0,+\infty \right)$ .


(4) $$L_{WIoUv3}=rL_{WIoUv1},r=\frac{\beta }{\delta \alpha^{\beta -\delta }}$$

Where β denotes the degree of outlier presence, $L_{IoU}^{*}\;$ representing the separation of L_IoU_ from the computation graph, with a exponential running average of momentum denoted by *m*, while *α* and *δ* serve as two hyperparameters.

## 5 Experimental results and discussion

### 5.1 Experimental platform and parameter settings

The experimental platform and parameters utilized in this study are presented in **Table 2**.

**Table 2:** Experimental environment for research on soybean leaf disease identification.

Configuration	Configuration Name	More Information
Hardware Configuration	CPU	Intel(R) Xeon(R) CPU E5-2680 v4 @ 2.40GHz
	Running Memory	30GB
	GPU	NVIDIA GeForce RTX 3080
	Video Memory	20GB
Software Configuration	operating system	Ubuntu 20.04
	Python Version	Python3.8.16
	Deep Learning Frameworks	PyTorch1.13.1
	CUDA Version	11.6

The experimental input image size is set to 640 × 640 pixels, with a batch size of 32, a total of 150 epochs, an initial learning rate of 0.01, and the use of the SGD optimizer.

### 5.2 Evaluation metrics

To objectively evaluate the recognition performance of the model, this study employed various evaluation metrics including accuracy, precision, recall rate, PR curve, average precision (AP), and mean average precision (mAP). The calculations for each metric are presented in Equations 5-7:


(5) $$\;Accuracy\;=\frac{TP+TN}{TP+TN+FP+FN}$$

(6) $$recision\;=\frac{TP}{TP+FP}$$

(7) $$Recall\;=\frac{TP}{TP+FN}$$

where TP: True Positive; TN: True Negative; FP: False Positive; FN: False Negative.

### 5.3 Experimental results

#### 5.3.1 Kernel size experiment

An extensive receptive field enhances the capacity of a model to capture more structured information. The most prevalent method involves augmenting the network depth by stacking multiple small convolutions (e.g., 3 × 3 convolutions) to expand the receptive field. However, this method has limitations in enhancing the Effective Receptive Field (ERF) (Luo et al., 2016). In recent years, numerous studies have highlighted the effectiveness of large convolution kernels, including OKNet (Cui et al., 2024), RepLKNet (Ding et al., 2022), LKDNet (Luo et al., 2023), and LaKDNet (Ruan et al., 2023), which utilize large convolution sizes of 63 × 63, 31 × 31, 21 × 21, and 9 × 9, respectively, to enhance the Effective Receptive Field and improve the capacity of a model to capture global information. In this study, we conduct a performance comparison experiment on large convolution kernels to determine the optimal kernel size. The results are presented in **Table 3**.

**Table 3:** Results of the kernel size experiment.

Kernel_size	Precision(%)	Recall (%)	mAP@0.5(%)	Parameters/Mb	GFLOPS
9	91.1	89.2	95.4	3.18	10.2
21	90.7	90.4	95.7	3.21	10.5
31	91.4	89.9	95.6	3.31	11.8
63	91.5	90.3	95.8	3.44	13.5

In conclusion, this study selected a kernel size of 21, as it demonstrated near-optimal performance across key metrics, including precision, recall, and mAP50, while simultaneously maintaining a relatively low parameter count (3.21 MB) and computational overhead (10.5 GFLOPS). This selection strikes an ideal balance between performance and resource efficiency. In contrast, larger kernel sizes (e.g., 31 and 63) resulted in performance improvements but also led to a significant increase in computational complexity. Thus, the kernel size of 21 emerges as the optimal trade-off between computational efficiency and model performance.

#### 5.3.2 Loss function parameter adjustment experiment

To investigate the impact of α and δ in the WIoU loss function on model performance, parameter adjustment experiments were conducted on the modified network structure. The results are presented in **Table 4**. Utilizing WIoUv3 with α=1.4 and δ=5 yields optimal model performance, with an mAP of 96.9%, which represents improvements of 0.9%, 0.8%, and 0.7% over CIoU, WIoUv1, and WIoUv2, respectively, along with enhancements in both Precision and Recall.

**Table 4:** Effects of α and δ on model performance.

Model	IoU	Precision(%)	Recall (%)	mAP@0.5(%)
YOLOv8-DML	CIou	91.8	90.9	96
	WIou_v1_	91.7	90.7	96.1
	WIou_v2_	92.4	90.9	96.2
	WIoUv3(α=1.4,δ=5)	92.7	92.7	96.9
	WIoUv3(α=1.6,δ=4)	92.4	91.4	96.5
	WIoUv3(α=1.9,δ=3)	92.2	90.9	96

#### 5.3.3 Ablation experiment

To assess the specific contributions of the C2f-DWR module, MEFP, LSCD, and WIoUv3 loss functions to the model performance in YOLOv8-DML, an ablation experiment was devised. The experimental results are presented in **Table 5**.

**Table 5:** Ablation experiment results.

C2f-DWR	MEFP	LSCD	WIoUv3	Precision(%)	Recall (%)	mAP@0.5(%)	Parameters/Mb
–	–	–	–	90.5	89.4	95.1	3.01
✓	–	–	–	90.6	90.8	95.6	2.89
–	✓	–	–	90.7	90.4	95.7	3.21
–	–	✓	–	90.3	89.4	95.2	2.36
–	–	–	✓	91.7	91.7	96.1	3.01
–	✓	✓	–	90.6	90.7	95.6	2.56
✓	✓	✓	–	91.8	90.9	96	2.45
✓	✓	✓	✓	92.7	92.7	96.9	2.45

– means that this module was not used in this round of experiment, ✓ means that this module was used in this round of experiment.

As presented in **Table 5**, the incorporation of the C2f-DWR module notably enhanced the model’s feature extraction capability, resulting in a 0.5% increase in mean Average Precision (mAP), while concurrently achieving a slight reduction in the number of parameters. The Multi-scale Enhanced Feature Pyramid (MEFP) module contributed to a 0.6% increase in mAP, underscoring its efficacy in fusing multi-scale feature information. However, this improvement was accompanied by a 6.64% increase in the number of parameters. The introduction of the Lightweight Shared Convolutional Detection (LSCD) head led to a 21.6% reduction in parameter count, while maintaining stability in precision, recall, and mAP values. Upon the substitution of the WIoUv3 loss function, all performance metrics showed marked improvement, with mAP increasing by 1.0%, indicating that WIoUv3 more effectively optimizes the regression loss. The combined integration of the MEFP and LSCD modules into the baseline model yielded notable improvements in Precision, Recall, and mAP values. Importantly, the lightweight LSCD detection head effectively mitigated the parameter overhead introduced by the MEFP, optimizing both model performance and parameter efficiency. When all four strategies were simultaneously applied, the model achieved optimal performance across all indicators. Specifically, Precision, Recall, and mAP improved by 2.2%, 3.3%, and 1.8%, respectively, while the total parameter count was reduced by 18.6%. These results validate the efficacy of the proposed YOLOv8-DML model for soybean leaf disease identification in natural environments, demonstrating a significant reduction in model complexity while enhancing accuracy.

#### 5.3.4 YOLOv8-DML disease detection results

**Figure 8:**
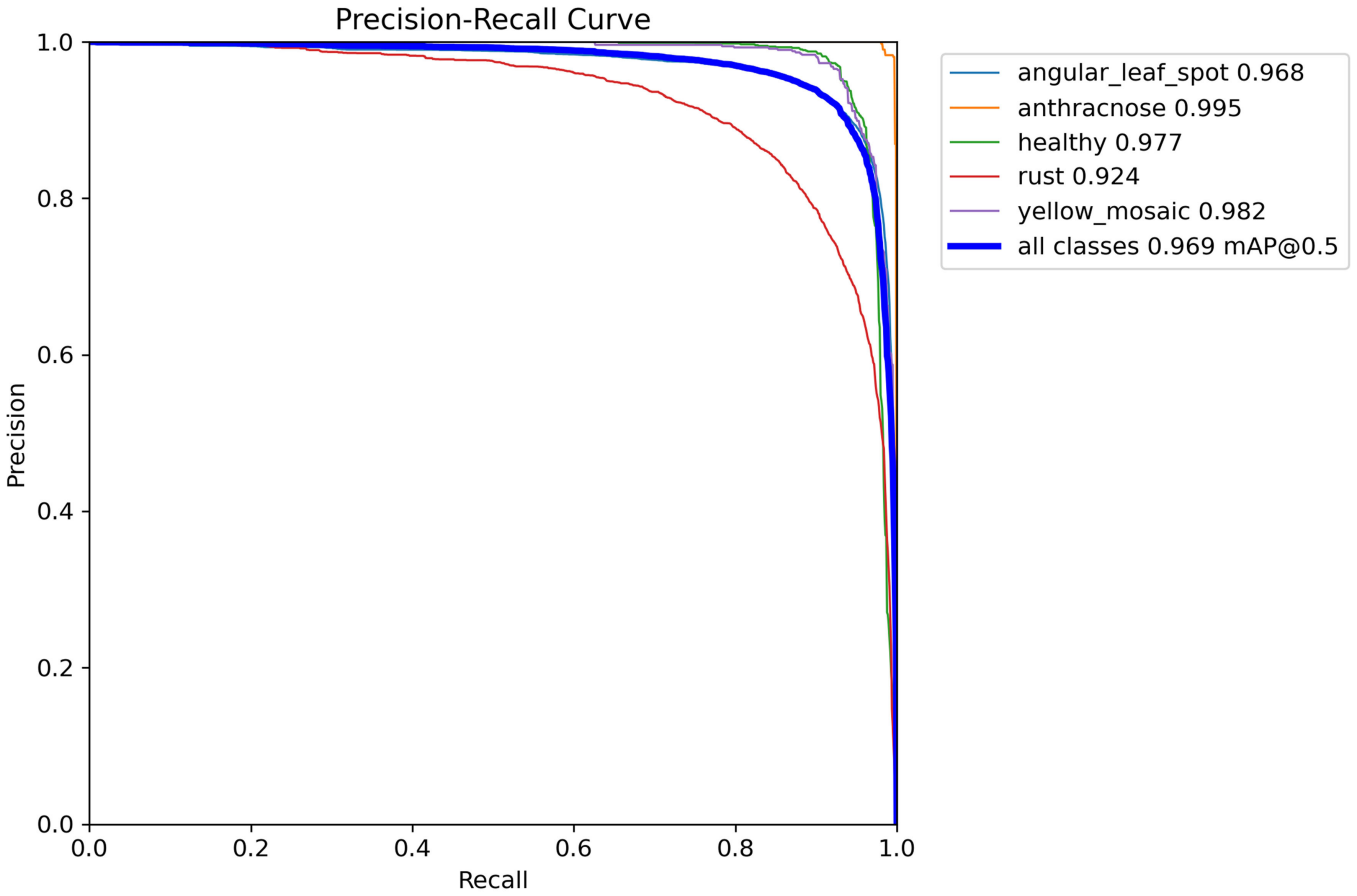
P-R curve of YOLOv8-DML model.

To rigorously evaluate the performance of the YOLOv8-DML model in detecting soybean leaf diseases, a simulation experiment was performed, with the resulting Precision-Recall (P-R) curve illustrated in **Figure 8**. The area under the curve represents the Average Precision (AP) across different disease categories.

As illustrated in **Figure 8**, the areas bounded by the P-R curves and coordinate axes for various soybean leaf disease categories show slight differences, suggesting that the YOLOv8-DML model demonstrates considerable robustness across multiple soybean disease types. The model achieved an average recognition accuracy of 96.9%, with soybean anthracnose displaying the highest accuracy at 99.5%, followed by yellow flower disease at 98.2% and rust at 92.4%. This discrepancy in rust recognition accuracy may stem from its initial lesions, which are smaller and subtler than those of other diseases, resulting in reduced feature representation during training, thereby affecting the precision of the model in detecting rust.

**Figure 9:**
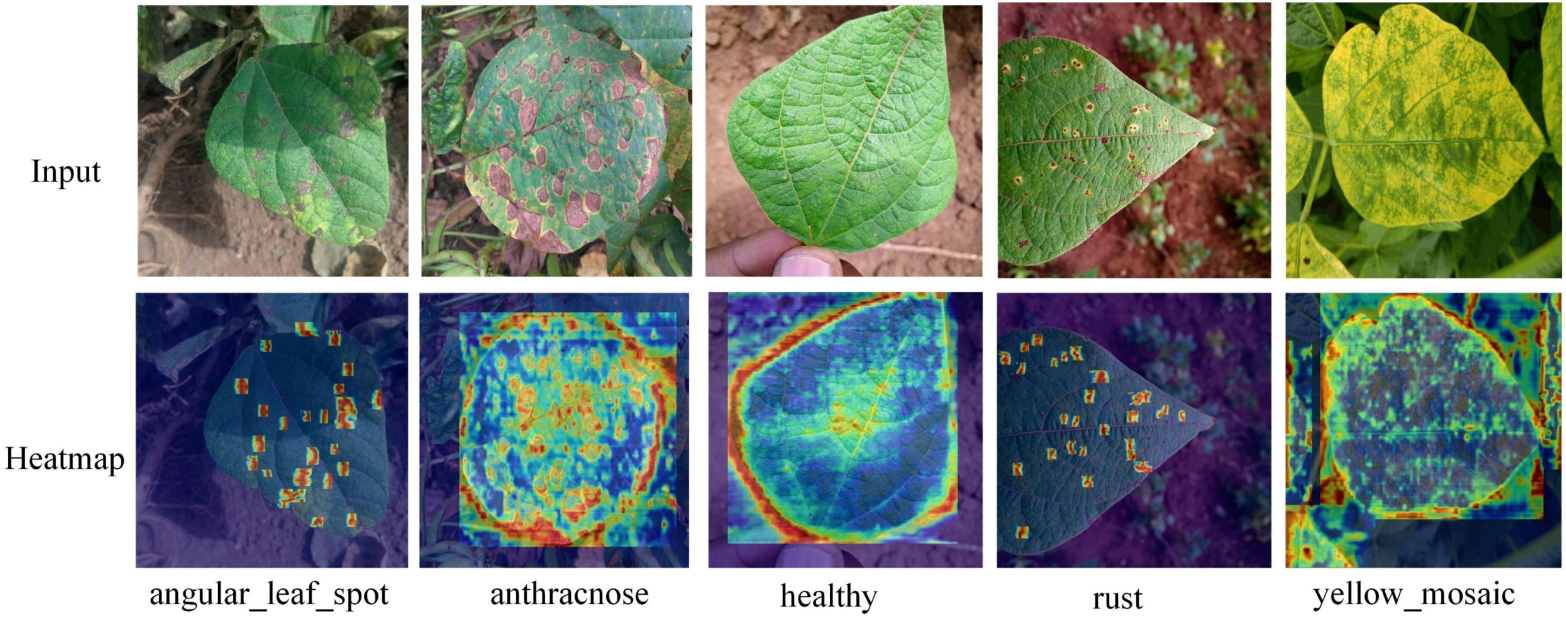
YOLv8-DML soybean leaf disease recognition heat map.

Using GradCAM++ heat map technology, the disease features generated by the YOLOv8-DML model were visualized and analyzed, as shown in **Figure 9**.

The GradCAM++ heat map technique is effective in identifying and capturing salient features associated with various diseases, providing a clear depiction of the model’s differential focus on disease characteristics across regions. As shown in **Figure 9**, regions with prominent features exhibit higher heat levels, visually representing the model’s attention to disease-relevant features.

#### 5.3.5 Performance comparison of different models

To comprehensively evaluate the disease recognition capabilities of the YOLOv8-DML model, this study conducted a comparative analysis of various mainstream object detection models on a soybean leaf disease recognition task. The comparative results are presented in **Table 6**.

**Table 6:** Experimental results of performance comparison of different models.

Models	Precision/%	Recall /%	mAP@0.5/%	mAP@0.5:0.95/%	Parameters/MB	GFLOPS
YOLOv7-Tiny	91	88.8	94.9	70.6	6.02	13.1
YOLOv8n	90.5	89.4	95.1	75.2	3.01	8.1
YOLOv9t	87.8	85.6	92.1	71.3	2.62	10.7
YOLOv10n	88.8	87.1	93.7	72.3	2.27	6.5
Efficient net	71.7	56.6	63.8	39.5	18.53	111.6
TOOD	93.7	92.5	96.8	77.9	32.03	125.9
RTMDet-Tiny	90.8	91.2	95.3	75.5	4.88	8.1
YOLOv8-DML	92.7	92.7	96.9	78.3	2.45	8.8

**Table 6** shows that although YOLOv8-DML’s parameter count and computational complexity marginally exceeded those of YOLOv10n, it achieved high precision and recall rates of 92.7% and exhibited exceptional performance in mAP@0.5 and mAP@0.5:0.95, scoring 96.9% and 78.3%, respectively, outperforming all comparative models. In comparison to the widely adopted YOLOv7-Tiny network in agricultural applications, YOLOv8-DML demonstrates superior performance across key metrics, including Precision, Recall, mAP@0.5, and mAP@0.5:0.95, with improvements of 1.7%, 3.9%, 2%, and 7.7%, respectively. Moreover, it achieves significant reductions in both parameter count and computational complexity, with decreases of 59.3% and 32.8%, respectively. Despite maintaining high detection accuracy, YOLOv8-DML operates with a parameter count of 2.45 MB and a computational complexity of 8.8 GFLOPS—both of which are considerably lower than those of more complex models such as EfficientNet and TOOD. Comparative experiments highlight that YOLOv8-DML effectively reduces computational load through its lightweight design and multi-scale feature fusion, thereby sustaining high recognition efficiency. This makes the model particularly suitable for deployment in resource-constrained environments. Furthermore, while TOOD exhibits slightly higher precision, its substantial computational cost and increased parameter count limit its scalability for real-world applications. In summary, YOLOv8-DML shows significant potential for practical use in soybean leaf disease recognition, offering a promising balance of high performance and computational efficiency.

#### 5.3.6 Comparison of recognition effects of different models

**Figure 10:**
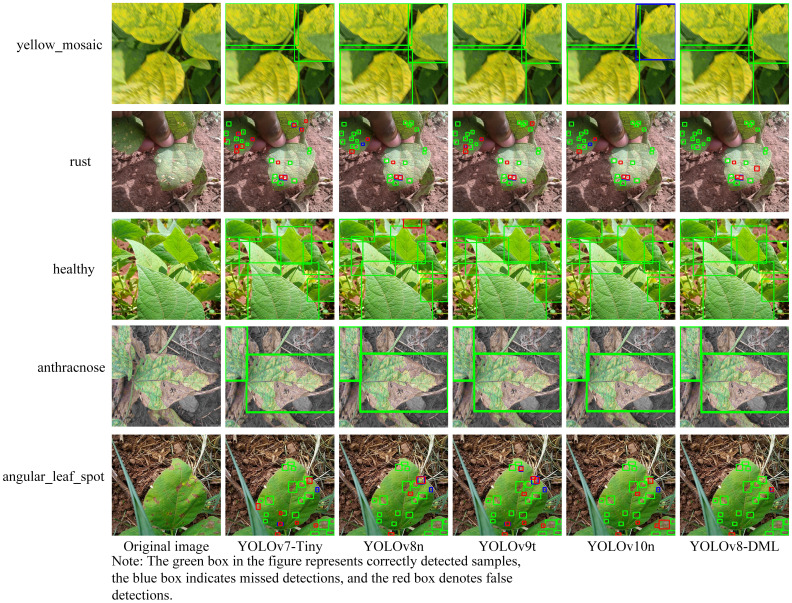
Dentification effect of soybean leaf diseases by different models.

To further validate the recognition performance of the YOLOv8-DML model for soybean leaf diseases in natural environments, this study compared and analyzed the model’s recognition outcomes against four other lightweight models: YOLOv7-Tiny, YOLOv8n, YOLOv9t, and YOLOv10n, using soybean leaf images captured under natural conditions. The experimental results are shown in **Figure 10**.

As illustrated in **Figure 10**, the five models effectively achieved accurate localization and identification of soybean leaf diseases with distinct characteristics such as anthracnose, healthy leaves, and yellow mosaic. Conversely, in the case of angular leaf spots and rust, which are diseases characterized by small lesions, diverse shapes, and subtle features, YOLOv7-Tiny, YOLOv8n, YOLOv9t, and YOLOv10n exhibited a higher incidence of false detections and a limited number of missed detections. In contrast, the YOLOv8-DML model demonstrated significantly superior performance compared to the other four models. The majority of diseases can be accurately detected, with the exception of a few instances involving leaf edges and extremely small or occluded lesions, which may be misidentified or overlooked. These findings indicate that the YOLOv8-DML model possesses a robust capability to effectively locate and identify small target lesions, suggesting its efficacy in detecting soybean leaf diseases in natural environments.

Additionally, we have incorporated a table-based comparison to clearly illustrate the differences between our proposed YOLOv8-DML model and other deep learning-based approaches in this field. The **Table 7** includes key factors such as dataset sources, environmental conditions, model architectures, feature extraction strategies, and recognition accuracy.

**Table 7:** Differences between the proposed YOLOv8-DML model and other deep learning-based methods.

Study	Dataset Source	Model	Environment	Small Lesion Detection	Multi-Scale Disease Handling	Loss Function	Accuracy (%)
Mohanty et al. (2016)	PlantVillage	AlexNet, GoogLeNet	Controlled	✗	✗	Cross-Entropy	85.2
Liu et al.(2024)	Self-collected	YOLOv4	Natural	✗	✓	IoU	88.6
Zhang et al. (2022)	Public dataset	YOLOv5	Mixed	✓	✗	IoU	90.5
Ours (YOLOv8-DML)	Public + Self-collected	YOLOv8-DML	Natural	✓	✓	WIoUv3	92.7

✗ means the model lacks or does not support the specified feature; >✓ means the model has or supports the feature.

## 6 Discussion

Our proposed model is designed with efficiency and adaptability in mind, making it suitable for integration into various agricultural monitoring platforms, including:

UAV-based monitoring: The lightweight nature of YOLOv8-DML, along with its improved small-lesion detection capabilities, makes it ideal for deployment on low-altitude UAVs for large-scale soybean field surveillance. This would enable real-time disease detection and precision agriculture applications, reducing the need for manual inspections.

Mobile applications: The model’s efficient architecture also allows for smartphone-based deployment, enabling farmers to use a mobile app to capture leaf images and receive instant disease diagnosis. This can help in early-stage disease detection and facilitate prompt decision-making in disease management.

Robotic-assisted disease monitoring: The proposed model can be embedded into autonomous agricultural robots equipped with high-resolution cameras, allowing for continuous disease monitoring and automated treatment recommendations.

The performance of the proposed YOLOv8-DML model when deployed on various platforms, including UAVs, mobile devices, and robotic systems, is an important consideration. While the current study primarily focuses on algorithmic advancements and validation under controlled experimental conditions, a theoretical analysis of the effects of camera height and motion speed on recognition accuracy is provided. At lower altitudes (e.g., 3m-5m), image resolution supports fine-grained lesion detection; however, at higher altitudes, the resolution decreases, potentially impacting accuracy. Similarly, slower camera speeds help maintain image clarity, whereas higher speeds lead to motion blur, which negatively affects performance. Practical deployment considerations include maintaining moderate altitudes and slow speeds for UAVs, as well as utilizing stabilized cameras for mobile and robotic platforms. Although specific experiments with varying camera heights and speeds are not conducted in this study, the design principles of YOLOv8-DML, such as multi-scale feature extraction and a lightweight architecture, indicate its potential for real-world applications. Future work will aim to validate these theoretical considerations through practical experimentation.

## 7 Conclusion

To address the challenges inherent in identifying soybean leaf disease spots in natural environments—challenges arising from significant variations in shape and size, as well as susceptibility to complex environmental interference—this study proposes the YOLOv8-DML model for soybean leaf disease identification. The model is built upon YOLOv8n and incorporates several key innovations. First, it introduces an expandable residual attention module and constructs the C2f-DWR module, which replaces the C2f components in the P4 and P5 layers of the network backbone, thereby significantly enhancing the model’s multi-dimensional receptive field. Subsequently, a multi-scale enhanced feature pyramid (MEFP) is implemented to improve object detection performance across multiple scales. Additionally, a lightweight detection head (LSCD) is designed to effectively reduce model complexity while maintaining performance. Finally, the loss function is modified to WIoUv3, which emphasizes the importance of small targets and low-quality samples, thereby improving detection accuracy.

Experimental results demonstrate that the mAP of the YOLOv8-DML model reaches 96.9%, reflecting a 1.8% improvement over YOLOv8n, while simultaneously achieving an 18.6% reduction in the number of parameters. When compared to other lightweight detection models within the YOLO series, YOLOv8-DML outperforms them in detection performance while offering a more optimized model size. Notably, the model’s advantages become particularly pronounced in the identification of dense small-target diseases, offering valuable insights for the detection of small disease targets in complex environments.

Despite the satisfactory identification performance of the YOLOv8-DML model for disease recognition, some challenges remain, including false detections and missed identifications, particularly at leaf edges, with extremely small lesions, and in occluded regions. The model primarily focuses on four-phase soybean leaves, and the diversity of soybean leaf diseases—resulting from the prevalence of various common diseases and healthy leaves—adds to the complexity. Therefore, further research is necessary to expand the disease dataset and enhance the generalization capabilities of the model. Future work will focus on the development of a mobile disease identification system. Specifically, we will continue augmenting the disease dataset and apply the developed model to identify additional plant diseases, validating its effectiveness and generalizability across multiple disease types. The improved YOLOv8-DML algorithm has broad applicability in real-world scenarios, from UAVs and mobile devices to robotic systems, which enable real-time disease detection. To further enhance real-world usability, future work will also consider the problem of model optimization in edge computing devices, including quantization and pruning techniques to reduce computational costs while maintaining high accuracy.

## Data Availability

The raw data supporting the conclusions of this article will be made available by the authors, without undue reservation.
